# Unveiling the Unexpected: A Case of Synergistic Drug Reaction to Cannabidiol

**DOI:** 10.7759/cureus.90992

**Published:** 2025-08-25

**Authors:** Aiman Balouch, Muhammad Osama Muslim, Muhammad Akram, Syed Saad Haseeb, Muhammad Naqash Chaudhary

**Affiliations:** 1 Internal Medicine, Northern Lincolnshire and Goole NHS Foundation Trust, Scunthorpe, GBR; 2 Internal Medicine, Capital International Hospital, Islamabad, PAK; 3 Acute Medicine, Northern Lincolnshire and Goole NHS Foundation Trust, Scunthorpe, GBR; 4 Internal Medicine, Hull University Teaching Hospitals NHS Trust, Hull, GBR

**Keywords:** acute hepatotoxicity, cannabis oil, drug-induced liver injury (dili), hepatic failure, herbal supplement adverse event

## Abstract

The widespread use of cannabis derived products has raised concerns with regard to their side effect profile, particularly hepatotoxicity. This case centers around a 45-year-old female patient receiving therapeutic cannabis oil, who demonstrated an acute surge in hepatic transaminases after excluding common etiologies, such as paracetamol and recent flucloxacillin, as both are hepatotoxic. A subsequent improvement in hepatic parameters following discontinuation reinforces the potential for an existing association between cannabis oil and liver injury. Furthermore, this case highlights the need for clinicians to recognize cannabinoid products as potential hepatotoxins, especially in patients with unexplained hepatic dysfunction. Given the growing access to such products, rigorous oversight and research have become essential in identifying potential health hazards and ensuring consumer safety.

## Introduction

Among the many reasons users have turned to the use of cannabis or cannabis derived products, such as cannabis oil, in recent years is its purported therapeutic benefits, like pain management, anti-inflammatory effects, and anxiety relief [1]. Cannabis oil, which is often used for its claimed properties, contains cannabidiol (CBD), a cannabinoid thought to have a relatively good safety profile. However, recent findings have raised concern among medical professionals about its potential hepatotoxicity [2,3].

Drug-induced liver injury (DILI) is a rare but serious side effect of cannabis oil, particularly when used alongside other medications. DILI is difficult to diagnose due to the challenge of linking suspected causal drugs to liver dysfunction [4]. Cannabis oil, a popular product for its various therapeutic uses, is emerging as a potential cause of liver injury, though little is currently known about its specific role in liver injury. Furthermore, the mechanisms behind cannabis oil-induced liver injury are not well understood, though it is thought to involve metabolic interactions, oxidative stress, and potential drug-drug interactions, especially when used in combination with other hepatotoxic substances.

In this case, the patient experienced abrupt liver failure following the use of cannabis oil, despite normal imaging. The liver injury was identified after ruling out common etiologies such as viral infection and autoimmune disease. This case underscores the importance of considering cannabis oil as a potential etiology of hepatic injury when other causes have been excluded, especially in the absence of clear diagnostic indicators. Although cannabis oil-related liver injury is uncommon, it should be considered in the differential diagnosis of acute liver injury, particularly in patients who use multiple medications or have unexplained liver dysfunction. Moreover, we advocate for enhanced pharmacovigilance to closely monitor the hepatotoxic potential of cannabis-derived products as their use continues to grow.

## Case presentation

A 45-year-old woman presented to the emergency room with severe exhaustion and slight discomfort in the right upper quadrant. Examination was unremarkable, and vitals were stable. The patient denied having used illegal substances in the past or alcohol use.

Drug history was significant for a five-day course of flucloxacillin (1 gm four times a day) that she had completed for infection, two weeks prior to this presentation. She had a documented history of new breast cyst pain, for which she had been treated with a combination of over-the-counter sporadic dosages of paracetamol (about 1-1.5 grams at the time of her presentation), continuously for a period of two weeks and cannabis oil, 1 ml three to four times a day, for the past 14 days, until the date of presentation. Given that the patient's paracetamol level was 3 mg/L, which is categorized as a subtoxic level, the patient was admitted, and a workup was initiated, and all of her regular over-the-counter prescriptions were suspended.

**Figure 1:**
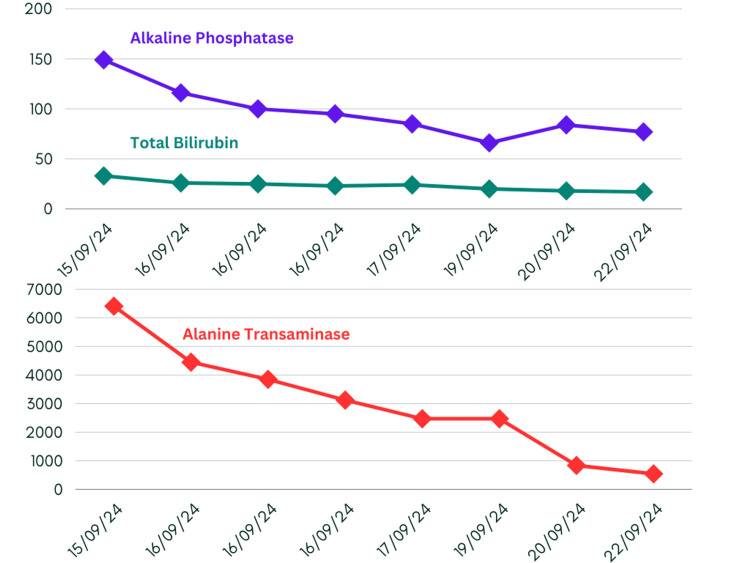
Trends in liver function tests

Laboratory blood investigation showed deranged liver synthetic and secretory functions. Initial studies revealed significantly raised liver parameters, including bilirubin levels at 33 μmol/L and alanine aminotransferase (ALT) levels rising past 6000 IU/L. Plotting of serial liver function tests revealed diminishing trends after initial conservative management (Figure 1). The flucloxacillin course was completed over two weeks ago, and hence, given the delayed onset, it is unlikely that flucloxacillin was the cause of her abnormal liver function tests (LFTs), as her ALT was significantly higher than alkaline phosphatase (ALP), making flucloxacillin-induced cholestatic liver injury less probable. The reference range of laboratory parameters are given in the Appendices.

**Figure 2:**
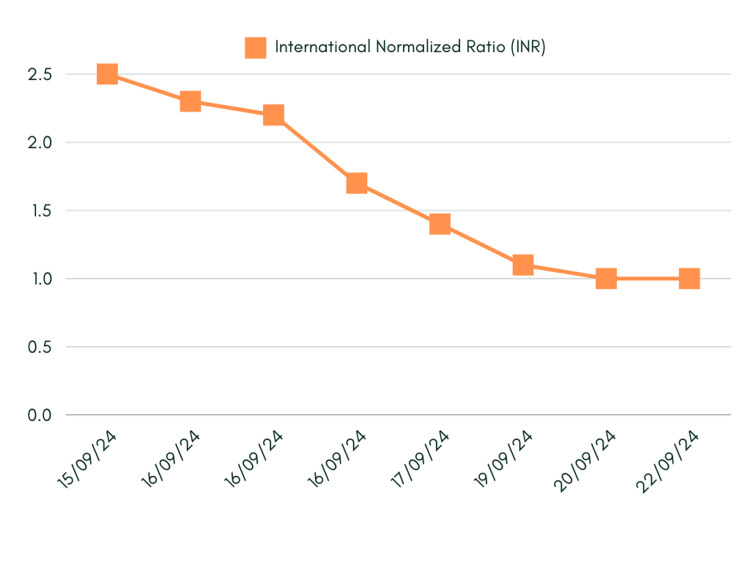
international normalised ratio (INR) trends

As noted in Figure 2, international normalised ratio (INR) levels likewise had a declining trend. 

**Figure 3:**
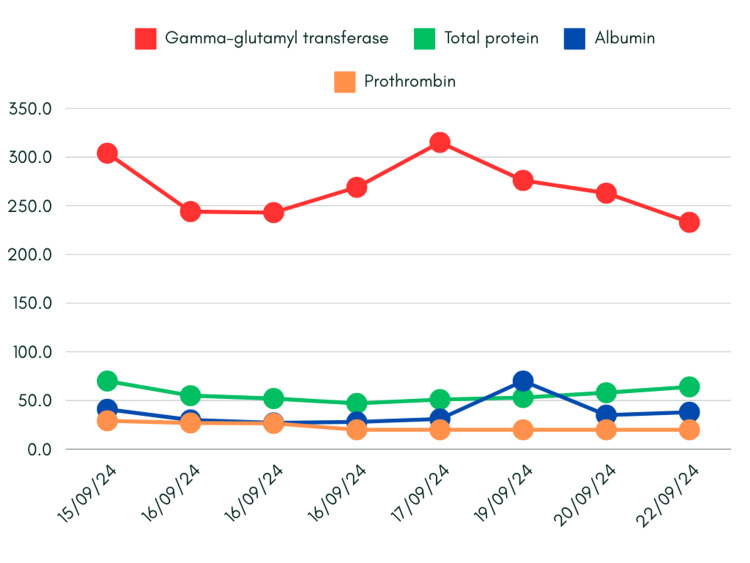
Trends in gamma-glutamyl transferase, total protein, albumin, and prothrombin levels

Albumin levels were found to be as low as 30g/l, while gamma-glutamyl transferase (GGT) was raised, but to a lower extent (Figure 3).

**Figure 4:**
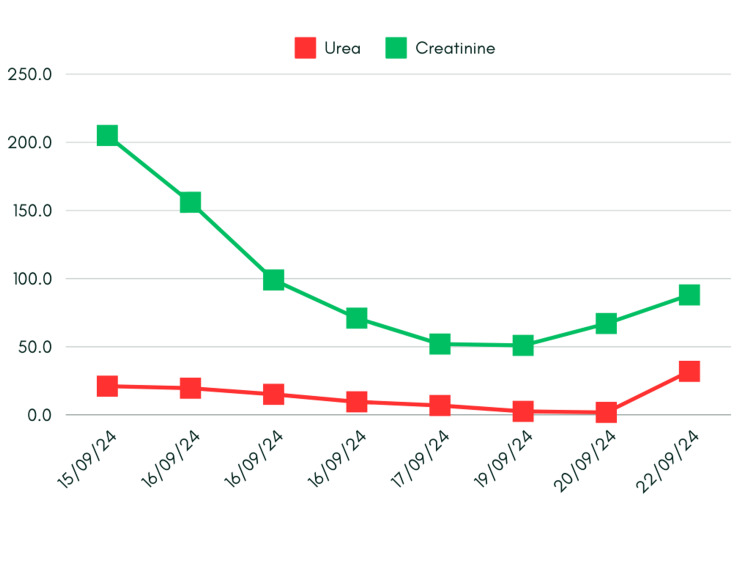
Trends in renal function tests

The test results also reveal declining urea and creatinine values at first presentation with potential improvements signifying a reversal in kidney function or unstable renal health (Figure 4). A comprehensive viral and autoimmune screen was performed to investigate other potential causes of liver injury. The C-reactive protein (CRP) was mildly elevated with no signs of infection. The viral serology results, including tests for hepatitis A, B, C, E, and Epstein-Barr virus, were negative, ruling out infectious causes. Additionally, autoimmune markers (antinuclear antibodies, anti-smooth muscle antibody, anti-LKM-1, anti-mitochondrial antibodies, anti-gastric parietal antibody) were negative, ruling out autoimmune hepatitis. Another relevant test, alpha-1 antitrypsin and ceruloplasmin level (0.15%), returned expected results, and no monoclonal band was detected on serum protein electrophoresis, which ruled out Wilson disease, alpha-1 antitrypsin deficiency, or multiple myeloma. Platelet was found to be normal.

**Figure 5:**
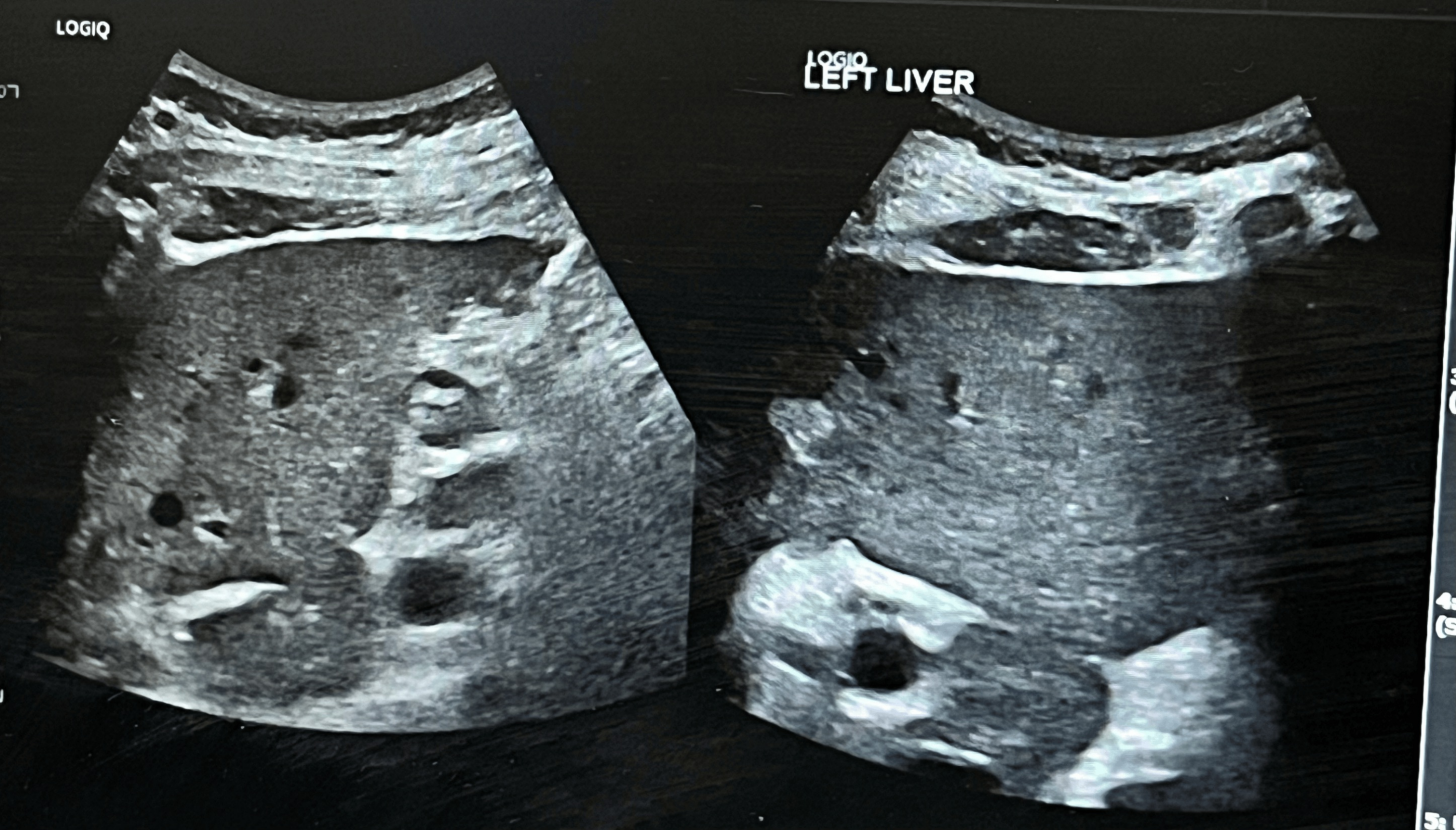
Ultrasound image showing no signs of cirrhosis or bile duct obstruction.

**Figure 6:**
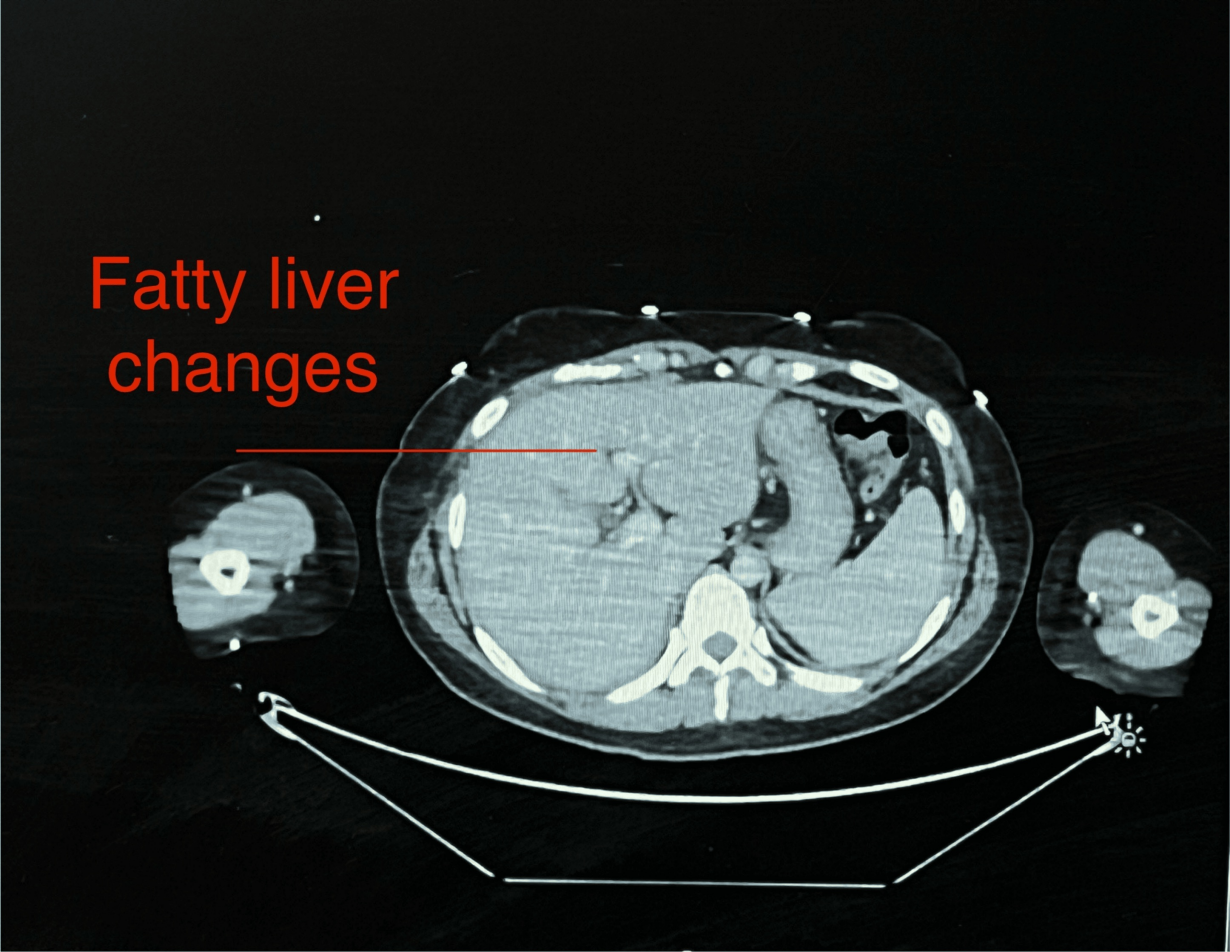
CT Images showing fatty liver .

Imaging studies were also performed, including an abdominal ultrasound and a contrast-enhanced CT scan to rule out cholestatic causes for abnormal liver functions. The liver ultrasound was unremarkable, showing no signs of cirrhosis or bile duct obstruction (Figure 5). The CT scan revealed mild fatty liver changes (Figure 6), which were deemed unlikely to explain the acute liver injury. No other significant liver pathology was identified on imaging.

Given the exclusion of other potential causes of drug-induced liver injury, CBD (cannabis oil) was considered the likely culprit, supported by R Factor scoring 12.3, which indicated the probable causality of liver injury. While cannabis oil is generally regarded as safe, there have been increasing reports of its potential hepatotoxic effects, mainly when used over a prolonged period or in susceptible individuals. The patient's liver injury was classified as idiosyncratic DILI, which is an unpredictable, dose-dependent adverse drug reaction. The discontinuation of cannabis oil was recommended, and the patient's liver function began to improve steadily after cessation.

Treatment primarily focused on supportive care, including liver function monitoring, the administration of intravenous fluid to address initial renal dysfunction, and suspension of regular medications. Regular monitoring of blood tests and vital signs was performed to assess the patient's response and ensure appropriate management. The patient showed a gradual improvement over her hospital stay, with the ALT level decreasing from 6408 on admission to 541 at discharge (Figure 1). Bilirubin levels normalized, and the INR returned to baseline 1.0. (Figures 1, 2). The patient was discharged with follow-up instructions to repeat the LFT in two weeks and a referral to the hepatology clinic for further follow-up. The patient was also advised to avoid cannabis oil and other potential hepatotoxic substances in the future.

## Discussion

The clinical evidence presented in this case suggested that cannabinoids administered concomitantly with other hepatotoxic substances may have augmented the risk and accelerated the progression of acute liver injury. However, further research is needed to definitively establish CBD oil as a contributing factor. While very few associations exist between cannabis oil and hepatotoxicity, our results suggest that cannabis oil is a critical co-factor for liver damage when used in conjunction with other drugs [5,6].

Several observations in the clinical course of the patient supported a synergistic hepatotoxicity hypothesis, where the concurrent utilization of cannabis oil, flucloxacillin, and paracetamol, each at a dose considered safe, resulted in severe hepatocellular injury, suggesting that the combination of these substances may have amplified the toxic effects rather than contributing independently to damage the liver. The biochemical pattern, with ALT levels greater than 6000IU/ml, indicated an amplification of the toxic effect rather than a simple additive injury. Additionally, the temporal relationship suggested that the liver abnormality occurred while the patient was using cannabis oil, in conjunction with the completion of a flucloxacillin course, indicating that the cannabinoids might prolong or exaggerate flucloxacillin's hepatotoxic potential. 

After a thorough literature search, we found that cannabinoids interact with different drugs, supporting Ben-Menachem et al., who found that cannabis and valproate increased stiripentol and ataxia-induced transaminitis in one patient [7]. Another study by Thai et al. gives some important pharmacokinetic evidence that cannabinoid (CBD) acts to inhibit CYP1A2 activity markedly, as was shown by 95% elevation in caffeine area under the curve (AUC) and prolonged elimination half-life when it's given concomitantly [8]. The hepatotoxic interactions observed in our case may be due to the potent inhibition of the key drug-metabolising enzyme, and many drugs actually known to be hepatotoxic (flucloxacillin, paracetamol, for example) require CYP1A2 clearance. Importantly, in the trial by Thai et al., six subjects had elevated liver transaminases, which adds support to CBD's propensity to cause liver injury by way of metabolic interference [8]. A systematic review and meta-analysis by Lo et al. demonstrated a significant association between CBD use and liver enzyme elevations and DILI, supporting the potential hepatotoxic effects of CBD [2].

These possible mechanisms are biologically plausible and may involve several pathways. Cannabinoids' ability to affect cytochrome P450 enzyme activity, in particular CYP3A4 and CYP2C9, which are highly important in the metabolism of flucloxacillin and paracetamol [9-11]. These metabolic interferences can result in increased levels of toxic drug metabolites, interference with normal detoxification pathways, and an increase in oxidative stress in hepatocytes. More recently, however, emerging research indicates that Δ9-THC and CBD may also directly intervene with hepatic mitochondrial function and promote apoptosis with attendant lowered threshold for DILI [12]. These mechanisms may have contributed to the liver dysfunction observed in our case study, despite the absence of classical risk factors, as CBD oil and its combination with other hepatotoxic materials possibly exacerbated the liver injury. However, the trends in bilirubin and albumin levels suggest that the hepatocellular failure was limited, despite the significant elevation of hepatic enzymes.

The administration of CBD-rich cannabis extract (CRCE) alongside acetaminophen to aged female CD-1 mice resulted in severe liver damage resembling sinusoidal obstruction syndrome, and it caused increased mortality rates together with enhanced activation of JNK signaling and oxidative stress pathways [13]. A report by Bass and Linz shows that CBD usage can cause unexpected liver damage alongside drug interaction effects [14]. The investigation conducted by Ewing et al. on male B6C3F1 mice demonstrated that acute and sub-acute CBD administrations caused hepatotoxic response by raising liver enzyme levels, increasing the weight of the liver, and generating substantial changes to stress-related genes and lipid regulatory genes [13]. Studies show that CBD leads to liver dysfunction effects primarily when patients consume the substance at elevated doses or maintain long-term usage [15,16].

Although cannabis is extensively used for medical benefits, it should not be ignored that these benefits do not negate serious potential adverse effects on both the liver and the nervous system functioning. The quick growth of cannabis product availability demands immediate regulatory control to establish standards for consistency, safety requirements, and usage guidelines [17]. Medical professionals must monitor patients who use cannabis products because these patients should be considered within the diagnosis process for unexplained liver harm and other potential toxic outcomes.

The medical evaluation of cannabis-induced liver damage faces difficulties because proper diagnostic standards are difficult to find. In the review by Eadie et al., medical staff concluded that DILI required exclusion methods to identify cannabis oil as the leading suspected origin of damage [18]. The clinical investigation and R Factor Scoring reveal the necessity of including cannabis oil diagnosis in excluding liver conditions that fail to have an identifiable etiology. This report highlights that while cannabis-induced hepatotoxicity remains rare, there is a possible correlation, underscoring the need for caution when prescribing pharmaceutical cannabis, particularly in persons with liver conditions or those with multiple diseases or multiple medications.

## Conclusions

This case study underscores the need for clinicians to consider over-the-counter CBD use in patients, emphasizing the significance of monitoring LLTs when managing multi-drug regimens. While cannabis-induced liver injury remains uncommon, the potential for drug interaction, particularly with hepatotoxic medicines, warrants careful pharmacovigilance. Further research into cannabis drug interactions is essential to better understand the hepatotoxic risks, especially as the use of cannabis products increases.

## Appendices

**Table 1:** Reference Range of Laboratory Parameters

Parameter	Unit	Normal Range
Urea	mmol/L	2.5-7.8
Creatinine	umol/L	45-84
Bilirubin	umol/L	0-21
Alanine transaminase	U/L	0-33
Alkaline Phosphatase	U/L	30-130
Gamma GT	U/L	6-42
C-reactive Protien	mg/L	0-5
Total Protein	g/L	60-80
Albumin	g/L	35-50
Platelet	10*9/L	150-400
International normalised ratio	1/1	0.9-1.1
